# Visualizing variation within Global Pneumococcal Sequence Clusters (GPSCs) and country population snapshots to contextualize pneumococcal isolates

**DOI:** 10.1099/mgen.0.000357

**Published:** 2020-04-30

**Authors:** Rebecca A. Gladstone, Stephanie W. Lo, Richard Goater, Corin Yeats, Ben Taylor, James Hadfield, John A. Lees, Nicholas J. Croucher, Andries J. van Tonder, Leon J. Bentley, Fu Xiang Quah, Anne J. Blaschke, Nicole L. Pershing, Carrie L. Byington, Veeraraghavan Balaji, Waleria Hryniewicz, Betuel Sigauque, K.L. Ravikumar, Samanta Cristine Grassi Almeida, Theresa J. Ochoa, Pak Leung Ho, Mignon du Plessis, Kedibone M. Ndlangisa, Jennifer E. Cornick, Brenda Kwambana-Adams, Rachel Benisty, Susan A. Nzenze, Shabir A. Madhi, Paulina A. Hawkins, Andrew J. Pollard, Dean B. Everett, Martin Antonio, Ron Dagan, Keith P. Klugman, Anne von Gottberg, Benjamin J. Metcalf, Yuan Li, Bernard W. Beall, Lesley McGee, Robert F. Breiman, David M. Aanensen, Stephen D. Bentley, The Global Pneumococcal Sequencing Consortium, Patrick E. Akpaka, Krow Ampofo, Houria Belabbès, Godfrey Bigogo, Abdullah W . Brooks, Philip E. Carter, Stuart C. Clarke, Alejandra Corso, Maria Cristina de Cunto Brandileone, Alexander Davydov, Idrissa Diawara, Sanjay Doiphode, Ekaterina Egorova, Naima Elmdaghri, Özgen Köseoglu Eser, Diego Faccone, Rebecca Ford, Paula Gagetti, Noga Givon-Lavi, Md Hasanuzzaman, Kristina G. Hulten, Margaret Ip, Aurelie Kapusta, Rama Kandasamy, Tamara Kastrin, Jeremy Keenan, Pierra Y. Law, Deborah Lehmann, Jennifer Moïsi, Helio Mucavele, Michele Nurse-Lucas, Stephen K. Obaro, Metka Paragi, Ewa Sadowy, Samir K. Saha, Eric Sampane-Donkor, Shamala Devi Sekaran, Sadia Shakoor, Shrijana Shrestha, Anna Skoczynska, Soo Ko, Somporn Srifuengfung, Peggy-Estelle Tientcheu, Leonid Titov, Paul Turner, Yulia Urban, Jennifer Verani, Elena Voropaeva, Nicole Wolter

**Affiliations:** ^1^​ Parasites and microbes, Wellcome Sanger Institute Hinxton, UK; ^2^​ Centre for Genomic Pathogen Surveillance, Wellcome Genome Campus Hinxton, UK; ^3^​ Big Data Institute, Li Ka Shing Centre for Health Information and Discovery, University of Oxford, Oxford, UK; ^4^​ Vaccine and Infectious Disease Division, Fred Hutchinson Cancer Research Center, Seattle, WA, USA; ^5^​ Faculty of Medicine, School of Public Health, Imperial College London, UK; ^6^​ Department of Veterinary Medicine, University of Cambridge, Cambridge, UK; ^7^​ Division of Pediatric Infectious Diseases, Department of Pediatrics, School of Medicine, University of Utah, 295 Chipeta Way, Salt Lake City, UT, 84108, USA; ^8^​ University of California Health, Oakland, CA, USA; ^9^​ Christian Medical College, Vellore, India; ^10^​ National Medicines Institute, Division of Clinical Microbiology and Infection Prevention, Warsaw, Poland; ^11^​ Fundação Manhiça / Centro de Investigação em Saúde da Manhiça (CISM), Maputo Mozambique, Instituto Nacional de Saúde, inistério de Saúde, Maputo, Mozambique; ^12^​ Central Research Laboratory, Department of Microbiology, Kempegowda Institute of Medical Sciences Hospital & Research Center, Bangalore, India; ^13^​ Center of Bacteriology, Adolfo Lutz Institute, São Paulo, Brazil; ^14^​ Instituto de Medicina Tropical, Universidad Peruana Cayetano Heredia, Lima, Peru; ^15^​ Department of Microbiology and Carol Yu Centre for Infection, The University of Hong Kong, Queen Mary Hospital, Hong Kong, PR China; ^16^​ Centre for Respiratory Diseases and Meningitis, National Institute for Communicable Diseases, Johannesburg, South Africa; ^17^​ Malawi-Liverpool-Wellcome-Trust, Malawi; ^18^​ NIHR Global Health Research Unit on Mucosal Pathogens, Division of Infection and Immunity, University College London, London, UK; ^19^​ WHO Collaborating Centre for New Vaccines Surveillance, Medical Research Council Unit The Gambia at The London School of Hygiene & Tropical Medicine, Fajara, The Gambia; ^20^​ The Faculty of Health Sciences, Ben-Gurion University of the Negev Beer-Sheva, Israel; ^21^​ Medical Research Council: Respiratory and Meningeal Pathogens Research Unit, University of the Witwatersrand, Johannesburg, South Africa; ^22^​ Department of Science and Technology/National Research Foundation: Vaccine Preventable Diseases, University of the Witwatersrand, Johannesburg, South Africa; ^23^​ Rollins School Public Health, Emory University, GA, USA; ^24^​ Oxford Vaccine Group, Department of Paediatrics, University of Oxford, and the NIHR Oxford Biomedical Research Centre, Oxford, UK; ^25^​ Queens Research Institute, University of Edinburgh, UK; ^26^​ Centers for Disease Control and Prevention, Atlanta, GA, USA; ^27^​ Emory Global Health Institute, Atlanta, GA, USA; ^28^​ See the full list of the The Global Pneumococcal Sequencing Consortium members, in acknowledgements

**Keywords:** *Streptococcus pneumoniae*, pneumococcal, whole genome sequencing, population structure, recombination, antibiotic resistance, pangenome, phylogenetic dating

## Abstract

Knowledge of pneumococcal lineages, their geographic distribution and antibiotic resistance patterns, can give insights into global pneumococcal disease. We provide interactive bioinformatic outputs to explore such topics, aiming to increase dissemination of genomic insights to the wider community, without the need for specialist training. We prepared 12 country-specific phylogenetic snapshots, and international phylogenetic snapshots of 73 common Global Pneumococcal Sequence Clusters (GPSCs) previously defined using PopPUNK, and present them in Microreact. Gene presence and absence defined using Roary, and recombination profiles derived from Gubbins are presented in Phandango for each GPSC. Temporal phylogenetic signal was assessed for each GPSC using BactDating. We provide examples of how such resources can be used. In our example use of a country-specific phylogenetic snapshot we determined that serotype 14 was observed in nine unrelated genetic backgrounds in South Africa. The international phylogenetic snapshot of GPSC9, in which most serotype 14 isolates from South Africa were observed, highlights that there were three independent sub-clusters represented by South African serotype 14 isolates. We estimated from the GPSC9-dated tree that the sub-clusters were each established in South Africa during the 1980s. We show how recombination plots allowed the identification of a 20 kb recombination spanning the capsular polysaccharide locus within GPSC97. This was consistent with a switch from serotype 6A to 19A estimated to have occured in the 1990s from the GPSC97-dated tree. Plots of gene presence/absence of resistance genes (*tet*, *erm*, *cat*) across the GPSC23 phylogeny were consistent with acquisition of a composite transposon. We estimated from the GPSC23-dated tree that the acquisition occurred between 1953 and 1975. Finally, we demonstrate the assignment of GPSC31 to 17 externally generated pneumococcal serotype 1 assemblies from Utah via Pathogenwatch. Most of the Utah isolates clustered within GPSC31 in a USA-specific clade with the most recent common ancestor estimated between 1958 and 1981. The resources we have provided can be used to explore to data, test hypothesis and generate new hypotheses. The accessible assignment of GPSCs allows others to contextualize their own collections beyond the data presented here.

## Data Summary

The following resources are accessible via www.pneumogen.net/gps and Figshare, all links are documented in the Supplementary Material (available Fig. S1 in the online version of this article). Input and output files, and scripts used to run BactDating on the GPSC phylogenies are available on Figshare. Roary output files from the 13 454 genomes are available on Figshare. Visualizations of gene presence and absence and distribution of recombination blocks across the genome for each GPSC are hosted on Phandango, with the input files available on the Phandango Github [1]. Phylogenetic snapshots are available for each of the 12 countries with >100 isolates, paired with metadata, can be interactively viewed in Microreact (links in the Supplementary Material) [2]. Geographical distribution and other metadata can be interactively viewed in Microreact for each of the 73 common GPSCs (links in the Supplementary Material) [2]. GPSC alignments with recombination masked are available on Figshare. Raw fastq data, assemblies and annotations for 13 454 pneumococcal genomes were previously released [3] to the European Nucleotide Archive (ENA) as part of the Global Pneumococcal Sequencing project (GPS) with metadata and accessions in the Supplementary Material (Fig. S1). Raw fastq data and assemblies for pneumococcal isolates from Utah have been deposited in the ENA under the study accession PRJEB34550 and individual accessions are in the Supplementary Material (Fig. S1). The authors confirm all supporting data, code and protocols have been provided within the article or through Supplementary Material files.

**Significance as a BioResource to the community**

Analysis of the DNA of bacteria, such as the pneumococcus, can provide important insights into how they cause disease. Whole-genome sequencing is increasingly cost effective to comprehensively type bacteria, though the analyses of such datasets often require specialist training. We have taken the results of our analyses and visualize them in an interactive online format to make the results and interpretation more accessible. Any new pneumococcal genome can be easily assigned to Global Pneumococcal Sequence Clusters using PopPUNK with the GPS database, or via Pathogenwatch. Furthermore, we provide international descriptions of GPSC recombination profiles, gene content, estimated age of emergence and population snapshots of geographical regions to provide even greater context. Providing such bioinformatic output in interactive format makes data exploration easier, allowing dissemination of genomic insights into the wider community, and can be used as teaching tools. These resources could also facilitate cross-disciplinary research beyond the original aims of the project, for example mathematical modelling of resistance, serotype switching or geographical spread.

## Introduction

Bacterial typing at the subspecies level to determine the lineage to which a pathogen belongs and distinguish it from others, is an important activity in the study and surveillance of infectious disease. Characteristics such as resistance, geographical spread and association with disease are key features of interest. Understanding their prevalence and distribution across lineages is informative for understanding the role of population structure in pneumococcal disease epidemiology. With the advent of high throughput sequencing, nucleotide variation across the whole genome can be used to cluster genetically related isolates into lineages, which are then a starting point for more detailed analysis. We previously published a genome-derived definition of pneumococcal lineages based on an international collection of ~20 000 pneumococcal genomes using PopPUNK [3, 4].

The uptake and incorporation of DNA from the environment into the chromosome via transformation and homologous recombination are known to contribute more nucleotide variation than mutation in the pneumococcus [5]. The capacity for recombination varies between lineages and recombination events are unevenly distributed across the genome, with known hotspots in regions under high selection pressure such as the penicillin-binding proteins and the capsular polysaccharide locus [6–8]. Recombination events can be detected and recombination rates quantified, which can be visualized across the genome to further explore recombination dynamics [1, 9]. As well as being biologically interesting, recombination obscures the true phylogenetic signal of vertical descent and is important to account for. Identifying recombination using blocks of dense SNPs lends itself to groups of closely related strains, and therefore relies on robust definitions of lineages.

Recombination can introduce new alleles or new genes into the chromosome. The latter contributes to the pneumococcal pangenome: the complete complement of genes observed in the species. The pangenome is made up of a core set of genes found in all isolates and accessory genes, including some that confer antibiotic resistance, that are variably present across the species. The frequencies of individual accessory genes are suggested to be in an equilibrium that is key in determining population structure [10–12 ].

The pneumococcal population structure, specifically the lineages observed and the extent to which they are established is known to vary between geographic locations. This is a constraint on local population restructuring after the introduction of pneumococcal conjugate vaccines (PCVs) [3, 11–13]. Comparisons of the presence/absence and prevalence of lineages between countries is also facilitated by the international definition of GPSCs as opposed to dataset-specific designations. Identifying features of the population structure unique to a geographical location and then contextualizing them in the relevant GPSC gives an international perspective. It can give insight into the acquisition and geographical spread of a resistance determinant, the introduction of a lineage into one country from another, and serotype switch or loss of capsule observed in one location.

Dating events in the pneumococcal population structure such as the migration of a lineage or the acquisition of a clinically relevant feature is often useful. After the introduction of PCVs, some lineages were observed to shift from a vaccine serotypes to non-vaccine serotypes. Identifying the recombination events that result in serotype switch and dating it has often revealed that the recombination event and serotype variant was established before vaccine implementation and was subsequently selected [3, 14, 15].

Here we provide population snapshots of 12 countries representing four continents in Microreact [2] annotated with the GPSCs and clinically relevant metadata. We additionally provide the lineage snapshots and recombination and gene presence/absence profiles for 73 common GPSCs interactively in Phandango [1]. We also provide dated GPSC phylogenies to allow the date of genomic events or geographical introductions to be determined. We present examples of how these resources can be used to answer topical pneumococcal questions, with preliminary interpretation, and demonstrate how external data can be assigned to GPSC using Pathogenwatch to aid comparisons between datasets.

## Methods

DNA extraction and sequencing were described previously [3]. Briefly 13 454 pneumococcal isolates drawn from pneumococcal disease surveillance programs and/or carriage studies in 30 countries were sequenced on an Illumina HiSeq platform and assembled and annotated as part of the Global Pneumococcal Sequencing project (GPS) [3, 16]. Isolates were clustered into lineages named Global Pneumococcal Sequence Clusters (GPSCs) using assemblies as input for PopPUNK [4]. Serotype, sequence type (ST) and antibiotic susceptibility were previously derived from the genomes [3]. The 13 454 assemblies annotated using Prokka were used as input for Roary with default minimum 95 % percentage identity for blastp, with and without splitting paralogues, with the former presented in Phandango [17, 18].

### International GPSC snapshots

Lineage analyses were performed on 73 common GPSCs, representing 782 down to 22 isolates (Supplementary Material). Illumina reads from each isolate were mapped against previously prepared references for each lineage [3, 19]. In brief, where a public closed reference did not exist a high-quality illumina draft assembly was reordered against a complete *S. pneumoniae* genome ATCC 700669 [3 ], the reference genome accession numbers are provided in the Supplementary Material. The resulting alignment was used as input for Gubbins to identify recombination blocks and create recombination free phylogenies with RAxML [9, 20].

### Dating

Taxa dates were recorded in years and the month converted to decimal, in the absence of month data the mid-year value of 0.5 was added. We assessed any temporal signal using the recombination-free phylogenies. BactDating was used to date 73 common GPSCs using the output of Gubbins [21]. We ran the BactDating R package with three replicates and one with randomized tip date through MCMC chains of 100 000 000 generations sampled every 100 000 states with a 10 000 000 burn-in using the mixed gamma model [21 ]. The three replicate MCMC chains were deemed to have converged with Gelman diagnostic of approximately 1 for mu, sigma and alpha using the coda R package [22]. We then assessed temporal signal by comparing the first replicate model to a model ran under the same parameters but with randomization of the isolate dates with the modelcompare function of the BactDating package [21 ]. Finally, we assessed whether the effective sample size (ESS) on the first replicate model was greater than 200 using the effectiveSize function of the coda R package [22].

As a comparison to BactDating, we additionally ran Bayesian evolutionary analysis software beast v2.4.1 on the four sub-clades of GPSC3 [23]. We ran three replicate MCMC chains of 100 000 000 generations, with a 10 000 000 burn-in, that were sampled every 1000 states with the discrete gamma model of heterogeneity among sites, the relaxed clock model of nucleotide substitution with the Bayesian skyline tree prior. ESS were greater than 200.

### Population snapshots

For the country-based, species-wide analysis, fastq reads from each isolate were mapped using BWA against a complete *S. pneumoniae* genome ATCC 700669 (NCBI accession code FM211187) [19]. The pseudo-genome alignment was then reduced to variant sites using SNP-sites for phylogenetic tree construction using FastTree2 [24, 25]. The SNPs were then reconstructed on the tree [26, 27]. Country-based analysis was only performed on the countries with isolates >100 (*n*=12, Table 1).

**Table 1.** Population snapshots

Country	No. of isolates	Percentage IPD	Sampling years
South Africa	4615	63 %	1991, 2005–2014
The Gambia	1647	24 %	1993,1996–2014
USA	1584	100 %	1998–2009
Malawi	1304	43 %	1997–2015
Israel	1143	100 %	2005–2014
Peru	607	31 %	2006–2011
China	504	42 %	1995–2001, 2009–2017
Brazil	420	97 %	2008–2009, 2012–2013
Nepal	416	16 %	2005–2009, 2011–2014
Poland	189	100 %	2007–2013
Mozambique	167	100 %	2008–2010
India	114	97 %	2007–2010, 2013–2016

IPD, Invasive Pneumococcal Disease.

### External datasets

We included an externally sequenced dataset that was not included in the PopPUNK definition of GPSCs to demonstrate that GPSCs can be assigned to any pneumococcal genome. Seventeen serotype 1 isolates collected from children with invasive disease from Utah, USA between 1996 and 2011 were included. Among them, metadata was available for 14 isolates, of which 13 were associated with complicated pneumonia with empyema. They were whole-genome sequenced on an Illumina HiSeq 2500 platform with 125 bp paired-end reads at the Huntsman Cancer Institute at the University of Utah. The data was imported and assembled as previously described [16]. Assemblies were submitted to Pathogenwatch, which assigns pneumococcal serotypes using SeroBA, and GPSCs using PopPUNK and the GPS reference database [3, 4, 28, 29]. Raw data and assemblies were deposited in the ENA under the study accession PRJEB34550. The isolates from Utah were also mapped to the GPSC31 reference (GenBank accession GCA_901234765) and combined with GPS GPSC31 strains to produce an alignment. Recombiantion was masked with Gubbins to build a phylogenetic representation of the external data and GPSC31 [9].

## Results and interpretation

### Assessing temporal signal in pneumococcal GPSCs

The BactDating models of temporal phylogenetic signal that had converged, were significantly better than the randomized dates model, and had effective population sizes of greater than 200, represented 70 % (51/73) of the GPSCs analysed. For the remaining GPSCs, 6/22 did not converge, 11/22 were no better than the randomized dates model, and for 5/22 the effective population sizes were <200 (Supplementary Material Fig. S1). Previously we reported recombination/mutation ratios for the GPSCs and calculated recombination-free pairwise SNP distances for the top 30 GPSCs [3]. We did not observe any extreme values for these metrics in the GPSCs we were unable to date, except smaller sample sizes and a lack of temporal signal.

**Fig. 1.**
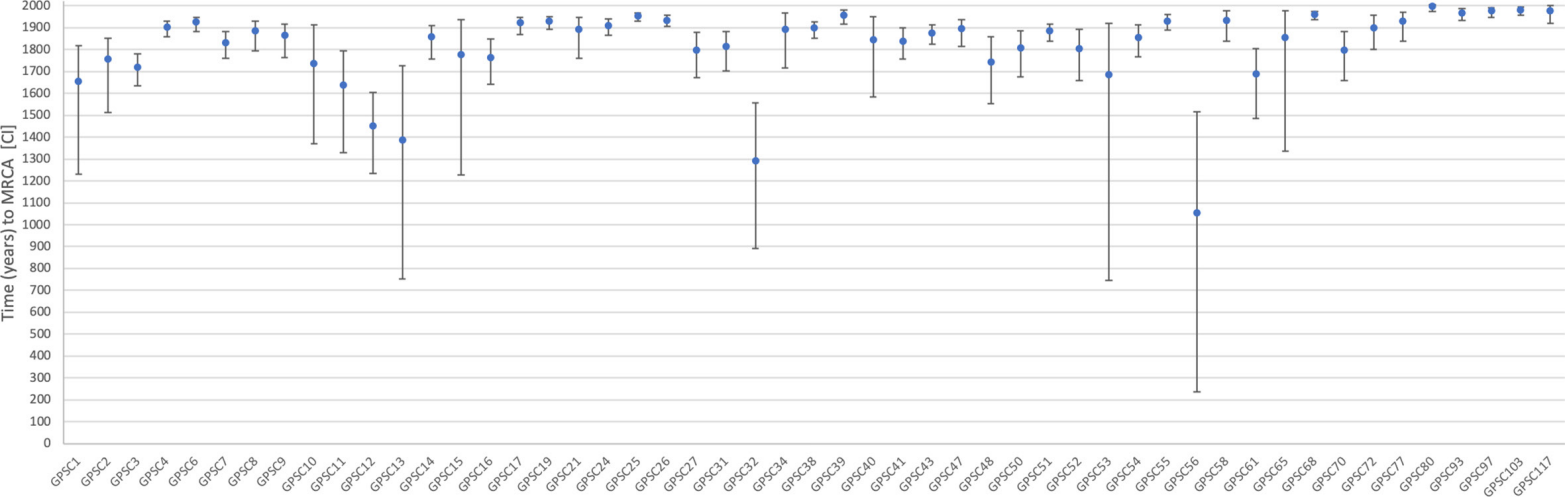
Point estimates of the year of the most recent common ancestor (MRCA) of each of the 51 GPSCs that could be reliably estimated. The 95 % confidence intervals (CI) are plotted.

The estimated dates across the lineages were somewhat consistent, with the average most recent common ancestor (MRCA) for the 51 GPSCs being 1814 [1640–1897] (Fig. 1). Large diverse lineages with strong sub-structure become prohibitively computationally intensive for dating using beast, comparing the most recent common ancestor for the four sub-clades CC53, CC62, CC100, CC1012 of GPSC3 from beast and BactDating revealed similar estimates (Table 2).

**Table 2.** Comparison of key feasible beast runs and BactDating models of phylogenetic dating

Lineage sub-clade	Year range	*N*	beast tMRCA	BactDating tMRCA
GPSC3	18	358	Run infeasible	1720.54 [1634.01–1780.92]
GPSC3-CC53	12	152	1879 [1820.49–1921.87]	1926.77 [1907.99–1941.56]
GPSC3-CC62	17	56	1912.82 [1667.10–1967.00]	1929.64 [1908.24–1944.03]
GPSC3-CC100	17	67	1937.13 [1920.19–1950.82]	1919.82 [1899.11–1936.45]
GPSC3-CC1012	17	83	1878 [1808.91–1925.02]	1840.70 [1802.97–1870.36]

CC, Clonal Complex; GPSC, Global Pneumococcal Sequence Cluster; tMRCA, Time to Most Recent Common Ancestor.

In general the GPSC dates are older but not inconsistent with previous reports of pneumococcal clones, which often represent sub-clades of GPSCs and where the definition of clone, sampling and geographical representation may not have been as extensive [30–32]. PMEN1 a sub-clade of GPSC16, was previously estimated to have shared a common ancestor in 1969 [1958–1977], the BactDating estimate of the MRCA of PMEN1 (ST81) isolates was identical 1969 [1958–1977] [14]. PMEN2 is a sub-clade of GPSC23 and was previously estimated to be of Western European origin between 1962 and 1974, the BactDating estimate of the MRCA of the PMEN2 (ST90) isolates of 1977 [1968–1983] overlaps with that estimate [31]. PMEN14 a sub-clade of GPSC1 was previously estimated to have shared a common ancestor in 1987 [1981–1991], the BactDating estimate of the MRCA of PMEN14 (ST236) isolates was much older and less certain 1885 [1807–1929]. Even when excluding the two most basal isolates of ST236 the estimate of 1949 [1904–1968] does not overlap with the previous estimate [6].

The conserved range of dates across GPSCs could result from two processes: (1) clones emerge and die at a roughly constant rate, meaning there is an underlying exponential distribution of ages; (2) a clone needs to be old enough to have to be measurably evolving and established enough to be well sampled, preventing younger clones from being dated. Many of these GPSCs represent globally spread clones, the period of human history from 1600 to 1800 is known as the proto-globalisation era, with large scale globalization beginning in the 1820s [33, 34 ]. This would have included the migration of families with children who may have been colonized with the pneumococcus, providing a new opportunity for global spread of local strains.

### Defining the pangenome

In 13 454 genomes, only 634 and 868 genes met the core gene definition of present in >=99 % or >=95 % of the collection respectively when paralogues were spilt. This is likely an underestimate as mis-assemblies, contig breaks and missing annotations can erode the number of genes that meet these criteria. The resultant core gene alignment was 544 759 bp long with 147 832 variable sites. A further 1957 genes were classified as shell genes (>=15 to <95 %) and 24 219 as cloud genes (>=1 to <15 %). The average number of genes defined as core (>=95 %) within lineages (GPSCs) was 1276. The mean core gene number per GPSC was higher than in the total collection in part because genes that are accessory to the species can be common to all isolates of a lineage, and because fewer genes will have been misclassified as non-core due to an accumulation of assembly and annotation errors.

### Example 1a: Exploring country phylogenetic snapshots using Microreact

**Fig. 2.**
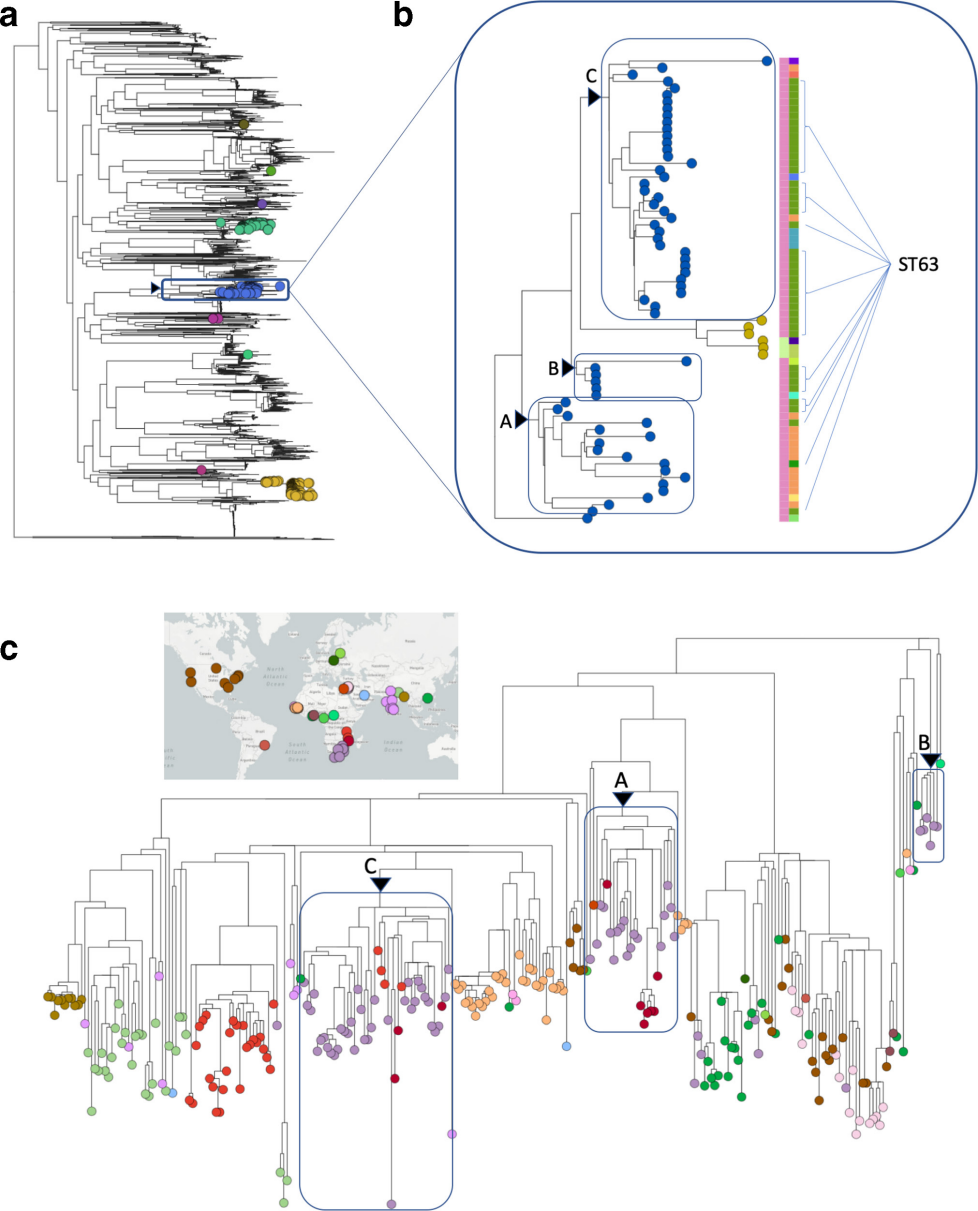
Contextualizing serotype 14 genotypes in South Africa. (a) Phylogeny of South African pneumococcal population structure, with taxa expressing serotype 14 highlighted by a coloured circle representing their GPSC assignment, GPSC9 (blue) is highlighted with a box. (b) Expanded South African GPSC9 subtree where taxa are coloured by serotype: 14 (blue) and 15A (yellow). The left metadata block is coloured by clonal complex: CC63 (pink), CC12576 (green). The right-hand metadata block is coloured by sequence type: ST63 (green), ST2414 (orange). Three sub-clades (A,B,C) that each contain at least one ST63 isolates are highlighted with a box. (c) The international GPSC9 collection have taxa coloured by country of isolation with a map based key, the three South African (purple) sub-clades (A,B,C) are highlighted in the international GPSC9 collection with a labelled box.

A phylogenetic snapshot of the population diversity in a single country can be clearly visualized interactively in Microreact. The interface is user-friendly and easy to filter on particular features (e.g. serotype), manifestation (carriage or disease), and/or demographic data (e.g. age). The South African population snapshot represents two cross-sectional colonization studies and national IPD surveillance surrounding PCV introductions. Such a dataset allows exploration of the population structure to contextualize numerous scenarios, for example it clearly highlights the diverse genetic background of serotype 14 isolates (Fig. 2a) [35]. The 278 South African isolates expressing serotype 14 in this collection were found within nine unrelated GPSCs – they do not share a common ancestor. Overall, 22 % (61/278) of serotype 14 isolates from South Africa were found in GPSC9 (61/67). They exclusively belonged to clonal complex (CC)63, and 38/61 (63 %) were ST63. However, there appeared to be phylogenetic structure within GPSC9/CC63/ST63 (Fig. 2a, b).

### Example 1b. Placing observations in the international context of a GPSC using Microreact

The context of the international GPSC9 phylogeny revealed three sub-clades in South Africa’s sample of GPSC9 serotype 14 ST63. Each sub-clade shared a common ancestor with isolates from other geographical regions before they share a common ancestor, suggesting they are not of shared South African descent (Fig. 2c). Using the dated GPSC9 phylogeny we can infer that the South African isolates represent three independently successful sub-clades established around the 1980s (A 1985[1976–1992], B 1995[1987–1999], C 1985[1978–1990]) and shared a common ancestor in the first half of the twentieth century 1933 [1909–1951].

### Example 2: Relating recombination events to changes in phenotype using Phandango

**Fig. 3.**
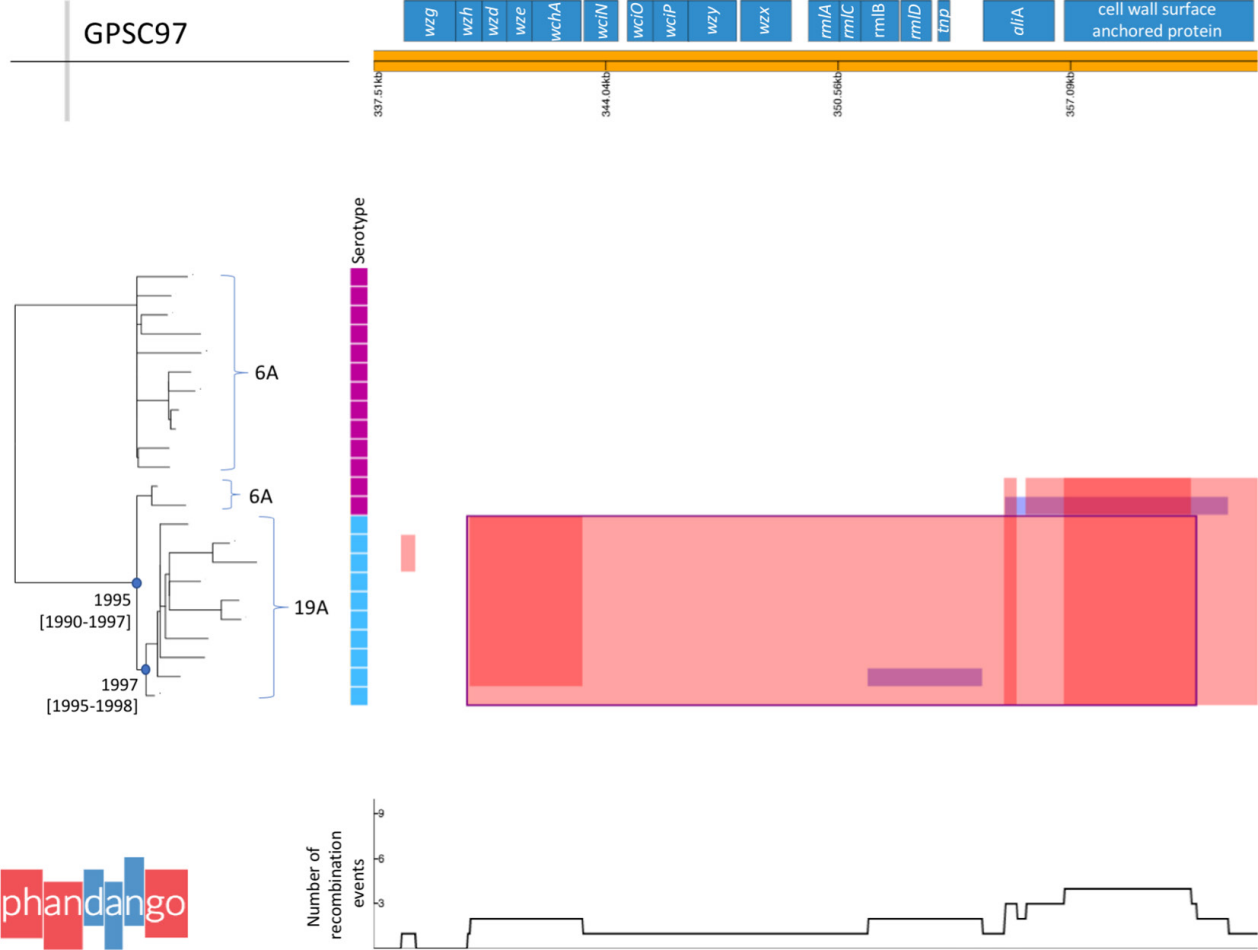
GPSC97 capsular polysaccharide locus recombination events. Phandango plot of recombination detected with Gubbins, focused on the cps locus, across the GPSC97 phylogeny. Isoaltes are annotated with their serotype in a metablock: 6A (pink) 19A (blue). Recombination blocks span the taxa in which they are detected and the region of genes affected in the reference. Red blocks affect *n*>1, blue blocks affect *n*=1. Overlapping blocks increase the density of the colour. A sliding window of the number of recombination events affecting any one position in the reference is plotted at the underneath. The major recombination block spanning most of the cps locus and common to all 19A isolates is consistent with the serotype switch, and is outlined in blue. The nodes for most recent common ancestors of the 6A and 19A isolates and the 19A isolates are designated with a blue circle, estimated dates and confidence intervals are given.

Recombination is known to facilitate clinically relevant changes in phenotype such as serotype switch. GPSC97 (CC1339/CC376), only observed in the USA in this collection, expressed either serotype 6A (13/23, 57 %) or 19A (10/23, 43 %). ST1339 was first reported expressing 6A and 19A in pubMLST in 2002 and 2006, respectively, in the USA. The earliest isolation date of 6A and 19A within GPSC97 in this collection was 1998 and 1999, respectively. There was phylogenetic structure to the serotypes expressed, and ancestral reconstruction of serotype suggests the clone originally expressed 6A. Visual inspection of recombination blocks in GPSC97 across the genome allowed the identification of a 20 495 bp recombination event spanning capsular polysaccharide locus (*cps*) genes *wzh* to *aliA*, which coincided with the change in serotype (Fig. 3). There was evidence of four other shorter recombination events affecting 1–12 isolates in the *cps* locus, which did not result in a change in serotype (Fig. 3). The date for the most recent common ancestor (MRCA) of the 19A strains was estimated to be 1997 [1995–1998], the MRCA for the 19A strains and the 6A strains was estimated to be 1995 [1990–1997] therefore the recombination event likely occurred between 1990 and 1998. This recombination event did not extend out to *pbp2X* and therefore had no affect on penicillin susceptibility. It was previously determined that all of GPSC97 had an identical *pbp2X* allele, and a predicted MIC of >=2 µg l^−1^ [3].

### Example 3: Detecting antibiotic gene acquisition associated with mobile genetic elements using Phandango

**Fig. 4.**
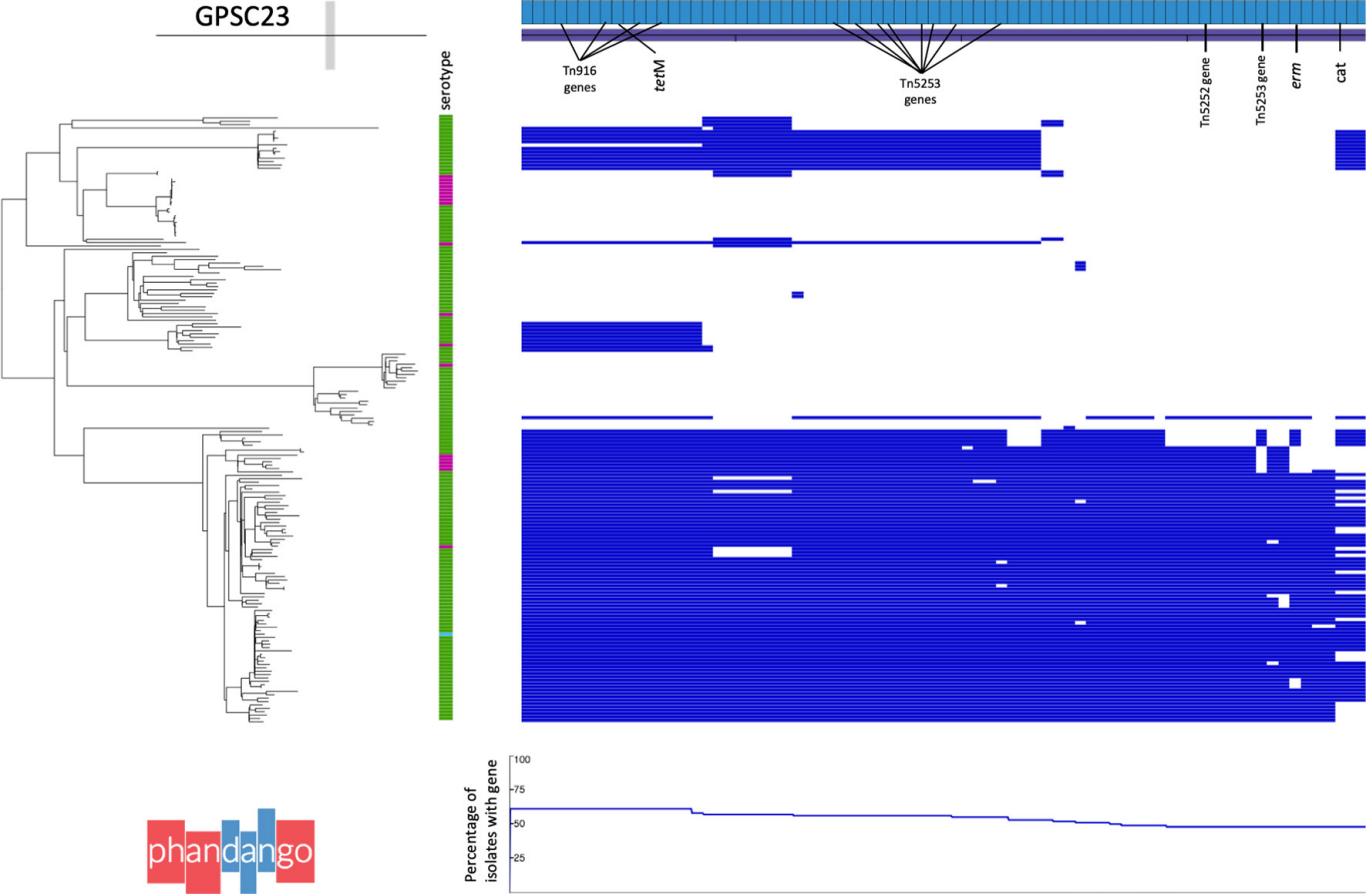
GPSC23 acquired resistance gene presence and absence. Phandango plot of Roary gene presence and absence, focused on the genes with prevalence and phylogenetic patterns similar to acquired resistance genes: *tetM* 61 %(110/180) *ermB* 43 %(77/180) and *cat* 40 %(72/180), across the GPSC23 phylogeny. Isolates are annotated with their serotype in a metablock: 6B (green), 6A (pink) and 19A (blue). Genes are shown as light blue bricks along the top and are sorted left to right by the proportion of isolates they are observed in. Presence (blue) and absence (white) of genes is plotted with respect to each isolates phylogenetic placement. A graph of the proportion of isolates the gene is observed in is plotted at the underneath.

Recombination and integration of mobile genetic elements (MGEs) can result in the acquisition of multiple accessory genes in a single event, for example multiple acquired resistance genes can be carried by a single MGE. Members of GPSC23 (CC273) were commonly (106/180, 59 %) predicted to be multidrug resistant (>=3 classes). This lineage is globally disseminated; observed in 13 countries representing Africa, Asia, Europe, North and South America. GPSC23 encompasses multiple Pneumococcal Molecular Epidemiology Network (PMEN) multidrug-resistant (MDR) clones: PMEN2 Spain^6B^ (ST90), PMEN17 Maryland^6B^ (ST384) and PMEN22 Greece^6B^ (ST273). This provides further confirmation on the relatedness of PMEN2 and PMEN22 reported previously [31]. Altogether, 33 % (59/180) of GPSC23 have the acquired resistance genes *cat*, *ermB*/*mefA* and *tet*M conferring resistance to chloramphenicol, erythromycin and tetracycline, respectively. The phylogenetic structure to the presence and absence of these genes in GPSC23 is variable (Fig. 4). The 58/59 isolates that carry *cat*, *erm*B and *tet*M fall in a sub-clade of 87 isolates encompassing PMEN2 and PMEN22. There appeared to be stable maintenance of *tet*M *n*=87/87, two independent losses of *erm*B *n*=76/87 and multiple independent losses of the *cat* gene *n*=58/87. Genes that are present in a similar proportion of isolates and similar phylogenetic pattern to these acquired resistance genes include those annotated as Tn*916*, Tn*5253* and Tn*5252* indicative of a composite transposon with gene loss since its integration (Fig. 4). Such variation is often difficult to assemble with short read data, the gene presence absence plot suggests a composite Tn*916*-Tn*5253* element accounts for most of the acquired resistance genes in this lineage. Using the dated tree for GPSC23 we estimate the date of acquisition to be around 1966 [1953–1975]. This fits all with previous work reporting inactivation or loss of resistance genes from a composite Tn5253-type integrative and conjugative element in PMEN2 in a similar timeframe [31].

### Example 4a: Contextualizing other datasets using the GPSCs and Pathogenwatch

Raw reads or assemblies can be dragged and dropped onto Pathogenwatch, which will QC the assemblies and check if they belong to the pneumococcus. Then it will assign their multi-locus ST, GPSC, serotype and report the detection of key genetic determinants of antimicrobial resistance (AMR). The assemblies from 17 serotype 1 isolates from Utah, which are external to the dataset used to define the GPSCs, were assigned by Pathogenwatch to GPSC31 (Table 3).

**Table 3.** Pathogenwatch output

	Summary
No. assemblies processed	17
No. analyses performed	102
Time taken	3 min
No. contigs	38–58
GC content	39.5–39.6 %
Assembly length	2.04–2.14 Mb
Species	* Streptococcus pneumoniae* (100 %, 17/17)
Serotype	1 (100 %, 17/17)
ST	ST227 (59 %, 10/17) ST306 (24 %, 4/17) ST304 (12 %, 2/17) ST4288 (6 %, 1/17)
Strain	GPSC31 (100 %, 17/17)
AMR determinants	None identified

AMR, Antimicrobial resistance; Mb, Megabases.

**Fig. 5.**
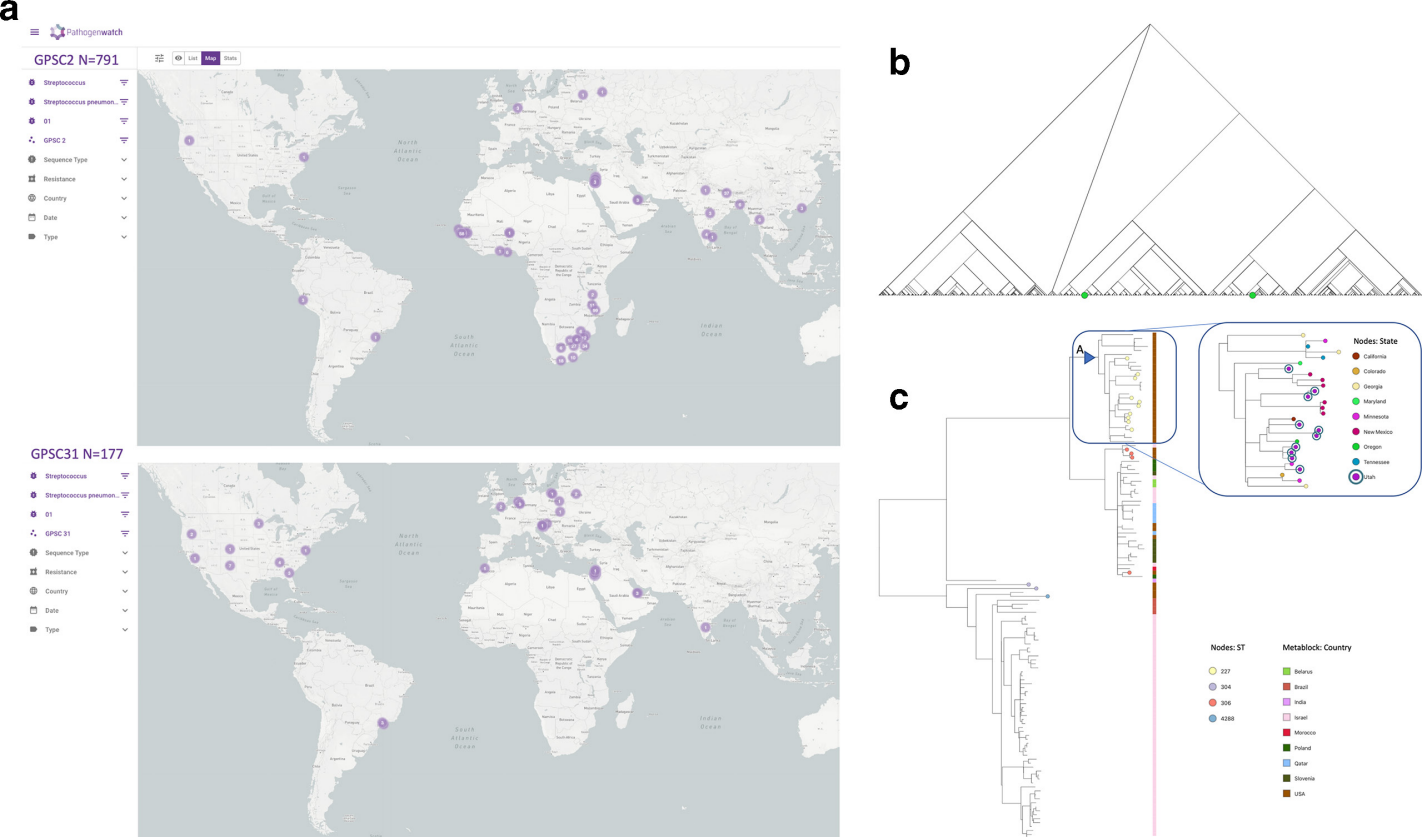
Giving context to serotype 1 isolates from Utah. (a) Different geographical distribution of serotype 1 genomes belonging to GPSC2 and GPSC31 on Pathogenwatch. (b) Serotype 1 isolates in the GPS project (*n*=893/13,454) fall exclusively into two lineages (green): GPSC2 (*n*=782) and GPSC31 (*n*=111), which are in different parts of the species-wide tree and do not share a recent common ancestor. (c) Utah isolates are highlighted on a and coloured by their ST (see key), the metablock shows the country of isolation across the GPC31 tree structure, and USA state on the expanded subtree. Triangle A denotes the most recent common ancestor 1973 [1958-1981] of the USA sub-clade in which the majority (10/17) of the Utah serotype 1 isolates were found.

The assignment of GPSCs to these isolates allows them to be put in a global context. All 20 027 pneumococcal genomes previously used to define the GPSCs are deposited in the Pathogenwatch [3]. Serotype 1 isolates in Pathogenwatch are only found in two lineages: GPSC2 and GPSC31, the database can be filtered on metadata, for example by strain (GPSC) and serotype, to view the geographical distribution, which differs between the two GPSCs (Fig. 5a). Differential geographical distributions of serotype 1 clones has been previously reported in the literature [36, 37]. Indeed within the GPS collection GPSC2 alone accounted for all the serotype 1 isolates from South Africa, Malawi and The Gambia, whilst GPSC31 was more common than GPSC2 in Israel and the USA, consistent with finding the Utah isolates only in GPSC31 [3 ].

These two lineages only express serotype 1 but do not share a recent ancestor in the species-wide tree (Fig. 5b). GPSC2 is composed of clonal complexes CC217, CC3581 and CC615, whilst GPSC31 includes CC5784, CC306 and ST227. ST227 was the predominant ST seen in children with pneumococcal empyema in Utah before conjugate vaccine introduction [38]. The relationship between these clones using genome-wide variation is consistent with previous designations of lineages A, B and C inferred from MLST where A represents GPSC31 and B and C are sub-clades of GPSC2 [36 ]. The tMRCA of the GPSC2 was estimated as 1655 [1230–1818] and for GPSC31 1814 [1702–1882], suggesting the serotype 1 CPS was transferred into a second lineage before the twentieth century.

The clones of serotype 1 have also been associated with different manifestations of disease, with lineage A (GPSC31) associated with pneumonia with or without empyema in Europe and North America and lineage B/C (GPSC2) with bacteremia and meningitis in Africa [37, 39, 40]. In Utah serotype 1 disease has been associated with pleural empyema and the STs of GPSC31 [38 ]. Serotype 1, 100 % ST 227, was identified in 50 % of children with empyema in Utah in the decade before the licensing of pneumococcal conjugate vaccine [41]. Serotype 1 was identified in 33 % of children with empyema after PCV7 introduction [38] but was relatively rare in other USA populations. Finally the absence of AMR determinants (Table 3) in the Utah isolates is typical of serotype 1, 72 % of GPSC2 and 99 % of GPSC31 were previously predicted to be pan-susceptible to the five antibiotics tested [3].

### Example 4b: Contextualizing additional data within a GPSC

After mapping the Utah isolates to the GPSC31 reference (GenBank accession GCA_901234765) and masking recombination using Gubbins we observed that 10/17 of the Utah isolates fell into a GPSC31 sub-clade of 28 isolates that were only isolated in the USA (Fig. 5c). These ten Utah isolates were spread across this sub-clade but GPS USA isolates were basal, this sub-clade in the dated GPSC31 tree shared a MRCA in 1962 [1941–1975]. The remaining seven isolates clustered in two other regions of the tree, which had broader geographical representation (Fig. 5c).

## Discussion

Large-scale bacterial genomic data generation is increasingly common in the next-generation sequencing era. The availability of raw data, assemblies and annotation currently deposited in the European nucleotide archive (ENA) are an invaluable open data resource with huge potential for research beyond the original research scope, especially if paired with metadata. However, visualizing results in an interactive tool can make the data vastly more accessible than the availability of primary data files [1, 2]. Constructing a bioinformatic workflow involving genotyping, identifying recombination and robustly building and dating phylogenies, is a specialized activity requiring resources and training. Automated genotyping from raw reads or assemblies combined with databases of genomes vastly increases accessibility, and the utility of private sequencing data in the context of public data processed in a standardized manner. Furthermore, interactive phylogenies of published data combined with metadata allows those who have yet to harness such bioinformatic skills to explore the output of genomic analyses and gain experience of its uses and interpretation.

Nonetheless, for those with sufficient bioinformatic experience, the interactive element is far superior to static figures and is invaluable for exploring the data to test and generate hypotheses. The interactive data that we present here, does still require knowledge of how to interpret phylogenetic trees and the limitations of bioinformatic methods. The tool Roary allows the rapid determination of pan genomes in large datasets from annotated assemblies. It relies on user-defined percentage identity thresholds, unsupervised algorithms for defining a gene, and assembly errors can lead to underestimation of core genes and a likely overestimation of singleton genes [17]. Gubbins readily identifies differences in SNP densities across the genome between isolates of the same lineage as recombination [9]. It relies on robust definitions of a lineage such as GPSCs, but is limited in its detection of recombination that does not result in a sufficient difference in SNP density, such as recombination within highly similar strains [9]. It does allow the identification of SNP dense recombination events that alter important phenotypes as we demonstrate here for serotype. Phandango allows such recombination data to be displayed interactively across the genome making recombination hotspots readily visible. Phylogenetic reconstruction relies on the vertical accumulation of variation, and therefore the identification of recombination and masking from lineage alignments is important. Many models currently exist that infer phylogenies with different advantages and disadvantages but all rely on the quality of the alignment [42]. Phylogenetic dating also depends on robust masking of recombination, the subsequent phylogeny, as well as sufficient temporal sampling and the presence of temporal signal. Dated trees allow us to add a temporal context to other observations such as capsular switch events, geographical dissemination and gene acquisition.

### Resource maintenance

Here we present phylogenetic analyses for 73 lineages and 12 countries, those that had reasonable sample numbers for such analyses. As the the GPS collection grows it will be possible to perform the analyses on further lineages and country population snapshots. Any resulting Microreact or phandango instances will be created and made accessible via the resources tab of the www.pneumogen.net/gps/. Adding further samples into existing phylogenies with ease and without repeating significant analyses is an area in which further bioinformatic development is needed. The definition of GPSCs will be updated, to include novel clusters as more sequence data is generated including those from external data if provided via www.pneumogen.net/gps/. Subsequent versions will be made available at www.pneumogen.net/gps/assigningGPSCs.html and incorporated into Pathogenwatch.

Microreact and Pathogenwatch are developed and maintained by the Centre for Genomic Surveillance (CGPS) at the Big Data Institute, University of Oxford and Wellcome Genome Campus, Hinxton, UK (http://pathogensurveillance.net). The underlying methods and software used to genotype rely on robust established schemes and databases, or bespoke pipelines and emerging software, with the latter two under continuous review and development to improve accuracy and reproducibly. CGPS have made comprehensive documentation available here: https://cgps.gitbook.io/pathogenwatch/. Those who wish to make use of Pathogenwatch to genotype their data (e.g. example 4a), or to visualize phylogenies representing their own data and metadata, with or without combining with GPS data in Microreact (e.g. example 4b) can do so privately by logging in. Any uploaded sequence data, phylogeny or associated metadata is not made available to other users or collaborators, or used in the internal analyses of the centre for Genomic Surveillence, without explicit consent. The Phandango website processes data ‘dragged and dropped’ in the users web browser with no data leaving the user's computer.

We show that the GPS collection of interactive results, dated phylogenies and GPSC definitions give useful insights for common research questions in the study of pneumococcus. This applies to exploring the GPS collection or external data. The data however also have use beyond the pneumococcal community, as a teaching resource, or for mathematical modelling of features relevant to other pathogens, to understand the evolution of antimicrobial resistance, geo-temporal spread of clones and the phylogenetic histories of populations.

## Data Bibliography

1. Gladstone RA, Lo SW, Lees JA, Croucher NJ, van Tonder AJ, *et al*. International genomic definition of pneumococcal lineages, to contextualise disease, antibiotic resistance and vaccine impact. *EBioMedicine* 2019;43:338–346.

## Supplementary Data

Supplementary material 1
